# Paradoxical Uterine Rupture and Fetal Survival in Placenta Accreta: A Case Report

**DOI:** 10.7759/cureus.71177

**Published:** 2024-10-09

**Authors:** Thomas J Curtis, Paul D Timmons, Demi J Blair, Richard P Smith, Thomas G Gray

**Affiliations:** 1 Obstetrics and Gynaecology, Norfolk and Norwich University Hospitals NHS (National Health Service) Foundation Trust, Norwich, GBR

**Keywords:** caesarean section, case report, hysterectomy, placenta accreta, uterine rupture

## Abstract

We describe a case of uterine rupture in the presence of abnormally invasive placentation, with fetal survival despite a long rupture-to-delivery interval (19.5 hours). The patient had previously had an intrapartum stillbirth delivered via caesarean section with an atypical uterine incision at 34 weeks’ gestation. She presented to a nearby hospital with abdominal pain at 25 weeks’ gestation, where an anterior uterine wall defect was diagnosed on ultrasound. A repeat ultrasound at our tertiary centre confirmed the uterine defect. With the patient reporting abdominal pain refractory to parenteral analgesia, we proceeded to delivery via laparotomy. The amniotic membrane and placental edge were visible on the abdominal entry, consistent with uterine rupture. The infant was born alive and transferred to the neonatal unit. Abnormally invasive placentation, later confirmed with histopathology, became apparent, and hysterectomy performed. We hypothesise that abnormally invasive placentation contributed to both uterine rupture and paradoxical fetal survival, as placental separation could not occur due to the placenta’s abnormal invasion.

## Introduction

We describe a highly unusual case of uterine rupture in the presence of placenta accreta, with fetal survival despite a long rupture-to-delivery interval (19.5 hours). Uterine rupture is estimated to complicate approximately one in 5,000 pregnancies, with the previous caesarean section the primary risk factor [1-3]. In uterine rupture, placental separation and fetal extrusion are the main contributors to perinatal mortality [4]. Placenta accreta spectrum (PAS) disorders occur in 11%-14% of pregnancies with placenta praevia and a background of one previous caesarean section [5]. We hypothesise that placenta accreta contributed to both uterine rupture and paradoxical fetal survival in this case, as placental separation could not occur due to the placenta’s abnormal invasion into the myometrium. The patient that this case describes has provided her consent for it to be published, including the figure, and reviewed the content for accuracy. This article was previously posted to the SSRN preprint server on September 12, 2024.

## Case presentation

This case is that of a patient in her mid-30s, in her third pregnancy, presenting at 25+0 weeks’ gestation reporting sudden-onset lower abdominal pain. Three years earlier, at another hospital, she had an intrapartum stillbirth at 34 weeks’ gestation following an unsuccessful operative vaginal delivery with forceps, attempted delivery of the fetus with vaginal incisions to the cervix and subsequent abdominal rescue delivery with a transverse uterine incision, extended upwards in the midline towards the fundus (inverted T incision). Prior to this, she had suffered a 13-week delayed miscarriage. The current pregnancy had been uncomplicated. Cervical length assessment with transvaginal ultrasound at mid-trimester showed no shortening nor funnelling. Ultrasound assessment at 23+1 weeks’ gestation showed a normally grown fetus and the placenta left lateral and low-lying.

On admission with abdominal pain to a neighbouring hospital without tertiary neonatal facilities, the patient was haemodynamically stable. Ultrasound assessment identified a defect in the anterior uterine wall with herniation of the amniotic sac into the peritoneal cavity. Fetal heart activity and growth were recorded as normal. A single dose of intramuscular 12mg dexamethasone to benefit fetal lung maturity and an intravenous magnesium sulphate infusion for fetal neuroprotection were administered. Ambulance transfer to our tertiary centre was arranged so that the patient was in a unit with capability to manage infants at extreme preterm gestations. On arrival, haemodynamic stability continued, but her constant lower abdominal pain persisted not relieved by parenteral opiate analgesia. The uterus remained soft on palpation, and there was no clinical evidence of peritonism.

**Figure 1:**
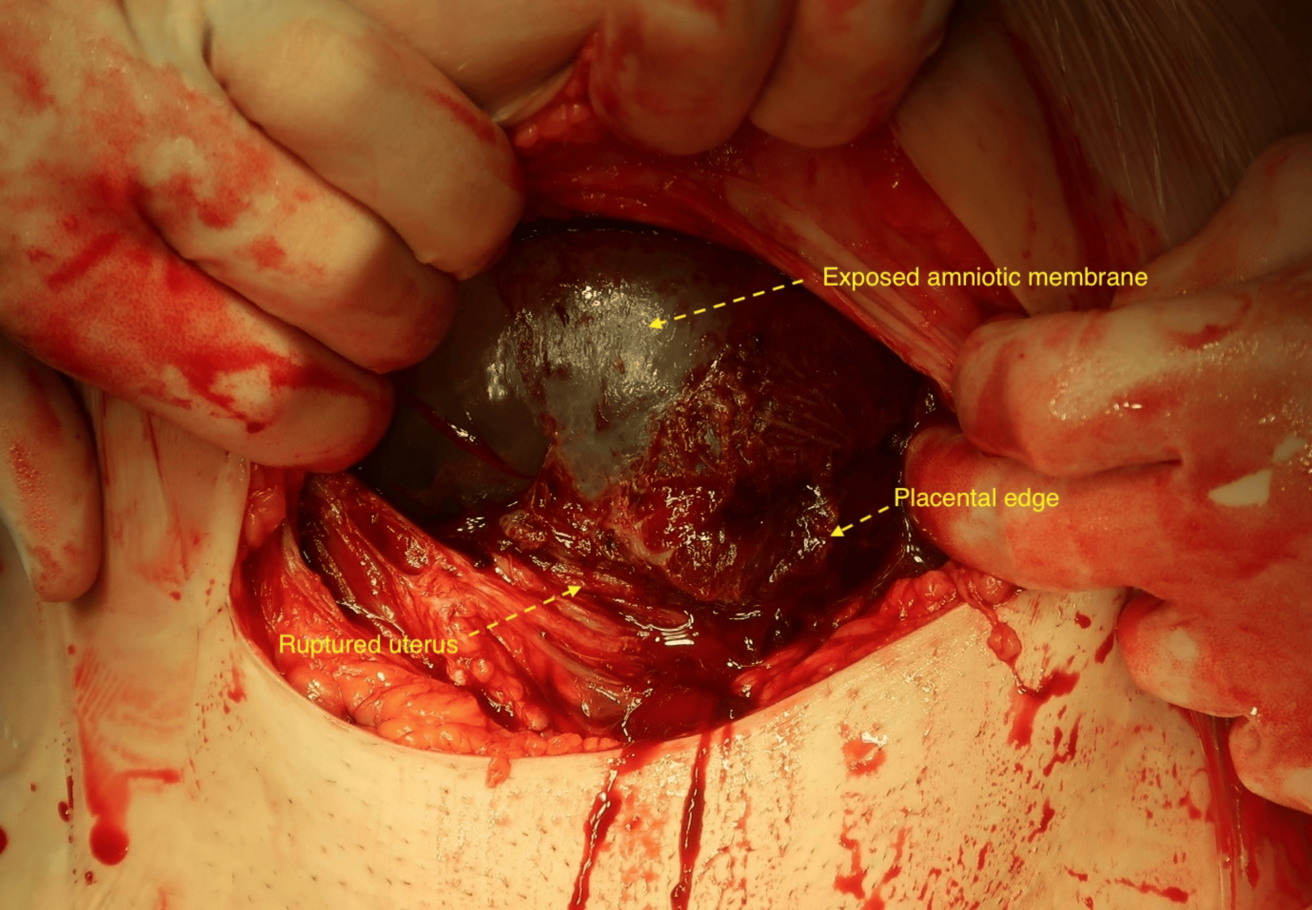
Exposed amniotic membrane and placental edge protruding through uterine defect; this was the view upon opening the abdominal cavity.

Point-of-care ultrasound assessment by the on-call obstetric consultant (a fetal medicine subspecialist) confirmed an 8cm (vertical) x 5cm (transverse) defect in the anterior uterine wall with herniation of the amniotic sac into the peritoneal cavity. Unfortunately, the setup of the portable ultrasound machine used did not allow for storage of these images. Ultrasound performed at 23 weeks had shown intact myometrium, implying this presentation to be an acute event, and taking account of the patient’s severe pain, a shared decision was made to proceed with caesarean delivery of the fetus, which was performed under combined spinal-epidural anaesthetic. Upon entry to the peritoneal cavity, a small (100ml) volume of blood was present; the amniotic membrane and placental edge were seen protruding from a complete uterine rupture (Figure 1). A live female infant was delivered in poor condition. Apgar scores at birth were one (one minute), seven (five minutes) and eight (10 minutes) with cord pH levels of 7.048 (arterial) and 7.144 (venous).

No evidence of placental separation was observed following birth despite administration of standard uterotonic therapy with oxytocin. It was not possible to identify a placental-myometrial interface, and controlled cord-traction was unsuccessful. Placenta accreta was therefore apparent, and a decision was made to proceed to hysterectomy, undertaken by the consultant gynaecologist on-call. Total estimated blood loss was 1,500ml, with four units of red blood cells transfused intraoperatively. Formal histopathological analysis demonstrated the placenta directly abutting the myometrium with no intervening decidua, consistent with diffuse placenta accreta.

The patient was transferred to the high-dependency unit postoperatively, where she remained for 24 hours. She made a good short-term recovery and was discharged home on the third postnatal day. On review at six weeks, she was further debriefed around her delivery but was well and making a positive recovery. Significant anaemia (Hb 100g/L) was observed in the neonate, suggestive of fetal haemorrhage prior to birth. The infant remained on the neonatal unit in the tertiary centre for five weeks and then spent six further weeks in their more local hospital neonatal unit before being discharged home at 36 weeks’ corrected gestation.

## Discussion

Uterine rupture is defined as a complete division of all three uterine layers (endometrium, myometrium and serosa) [6,7] and is estimated to complicate approximately one in 5,000 pregnancies [1-3]. It occurs in spontaneous labour in around 0.5% of those with a previous caesarean section but is much rarer outside of labour (<0.02%) [1]. Previous uterine surgery (usually caesarean section) is the primary risk factor, and it is hypothesised that this risk is increased further in the presence of an atypical uterine scar such as a midline or inverted T incision [2,3]. Patients attempting vaginal birth after caesarean section are advised to deliver in hospital with improved maternal and neonatal outcomes [1]. A shorter rupture-to-delivery interval is associated with improved neonatal survival with resultant placental separation and/or fetal extrusion cited as the primary contributors to perinatal mortality [4]. A paucity of data exists on the relationship between uterine rupture and PAS disorders, but it would seem logical that the pathophysiology of abnormally invasive placentation carries potential for gradual weakening of the scarred myometrium.

Other cases describing unexpected fetal survival in the context of uterine rupture have considered a phenomenon of "masked" uterine rupture [8-10]. In these cases, either a structure external to the uterus or a fetal part occluded the rupture and prevented the rupture cascade progressing. The case described here is distinct from this phenomenon, no occlusion of the rupture occurred. Instead, the co-existence of placenta accreta with the uterine rupture ensured fetal survival because the placenta was unable to separate.

## Conclusions

We believe this case to be noteworthy as fetal survival would not be expected following an acute uterine rupture of this magnitude and event-delivery interval (19.5 hours). We hypothesise that the co-existence of placenta accreta paradoxically contributed both to an increased risk of uterine rupture and to fetal survival; because placental separation could not occur, blood supply to the fetus was maintained. Likewise, PAS disorders may contribute to the weakening of the myometrium, increasing the risk of uterine rupture.
